# Structural analysis of clingstone and freestone peach (*Prunus persica* L.) plastome genome: provides insight into phylogeny and time diversification

**DOI:** 10.1016/j.jgeb.2026.100660

**Published:** 2026-02-28

**Authors:** Mokhtar Said Rizk, Alsamman M. Alsamman, Heba A.M. AbdAlla, Mohamed Abd. S. El Zayat, Ahmed H. Hassan, El-Shaimaa Saad El-Demerdash, Mohamed Z.S. Ahmed, Yuepeng Han, Mohamed Hamdy Amar, Achraf El Allali

**Affiliations:** a Genetic Resources Department, Desert Research Center (DRC), Cairo, Egypt; b Laboratory of Genomics and Genome Editing, South Sinai Regional Plant Gene Bank, Desert Research Center, Cairo, 11753, Egypt.; c Department of Genome Mapping, Molecular Genetics and Genome Mapping Laboratory, Agricultural Genetic Engineering Research Institute (AGERI), Agricultural Genetic Engineering Research Institute, Giza 12619, Egypt; d International Center for Agricultural Research in the Dry Areas (ICARDA), Giza, Egypt; e Botany Department, Agriculture and Biological Institute, National Research Centre, Giza, Egypt; f State Key Laboratory of Plant Diversity and Specialty Crops, Wuhan Botanical Garden of Chinese Academy of Sciences, Wuhan 430074, China; g Center for Converging Sciences and Emerging Technologies (CoSET), Benha National University, El-Obour City, P.O. 11828, Egypt; h Bioinformatics Laboratory, College of Computing, University Mohammed VI Polytechnic, Ben Guerir 43150, Morocco

**Keywords:** *Prunus persica*, Plastome genome, Freestone, Clingstone, Simple Sequence Repeats (SSRs), Plastome phylogenomic, Divergence time estimation

## Abstract

**Background:**

The complete plastome genome of peach represents a valuable genomic resource for elucidating the evolutionary history and phylogenetic relationships within Prunus species.

**Methods:**

This study performed plastome sequencing of two peach cultivars Clingstone (CLS) and Freestone (FRS) along with the wild relative *Prunus mira,* using the Illumina HiSeq 2500 platform. In this study, the plastomes of two peach cultivars—Clingstone (CLS) and Freestone (FRS)—and the wild relative *Prunus mira* were sequenced using the Illumina HiSeq 2500 platform. A broad comparative assessment was enabled by comparing these sequences with 22 previously sequenced Prunus plastomes.

**Results:**

All 25 plastomes exhibited a conserved quadripartite structure, with genome sizes ranging from 157,685 to 158,955 bp and an average GC content of 37.72 %. Structural variations were observed, including gene rearrangements and boundary shifts between the IR, LSC,and Small Single-Copy (SSC) regions. These boundary shifts, together with the identified sequence rearrangements, highlighted potential mutational hotspot regions. Considerable diversity was detected in Simple Sequence Repeats (SSRs), including polymorphic loci with total counts ranging from 559 to 1064 per plastome, comprising mono-, di-, penta-, and hexa-nucleotide motifs.Variable Simple Sequence Repeats (SSRs) and codon usage patterns were identified as evolutionary hotspots. Out of 40 genes, 28 single-copy and 12 multi-copy were consistently found across all plastomes. Such variation patterns underscore regions with high phylogenetic informativeness. Phylogenomic analysis revealed well-supported monophyletic clades, clarifying evolutionary relationships among peach, cherry, and almond groups. Divergence time estimates place the split between cultivated peach (*Prunus persica*, including CLS and FRS) and P. mira in the Eocene.

**Conclusion:**

This comprehensive plastome-based study enriches our understanding of the evolutionary dynamics of peach cultivars, particularly Clingstone and Freestone, in relation to wild Prunus relatives. The identified SSR loci, codon usage features, and structural variations offer potential molecular markers for peach genetic evolution and precise taxonomic resolution at the subspecies level.

## 1 Introduction

The plastome is indispensable for life, functioning as the key photosynthetic organelle in plant cells that converts light energy into ATP and carbohydrates. The availability of sequenced plastomes has significantly advanced our understanding of their biology and genetic basis^1^. Studying plastomes offers valuable insights into intracellular gene transfer, as well as genomic conservation and diversity. Furthermore, plastome transgenes can be engineered to improve crop traits and facilitate the production of high-value compounds for agricultural and medical applications.^2^

Plastome sequencing has created new opportunities to explore the role of plastid genes in plant adaptation and evolution. The plastome contains important genes involved in various biological processes^1^. Its highly conserved organization and gene composition make it particularly valuable for molecular investigations, such as evolutionary and population genetic studies, and for rapid species identification via DNA barcoding.^3^, ^4^, ^5^ Moreover, next-generation sequencing (NGS) technologies enable the identification of chloroplast-derived markers, including simple sequence repeats (SSRs), single-nucleotide polymorphisms (SNPs), and insertions/deletions (InDels). These SSRs are predominantly located in noncoding regions, particularly within intergenic spacers.^6^

Peach (*Prunus persica* L.) is a widely cultivated species classified under the Rosaceae family, which includes over 3,000 species with a wide range of architectural forms, evolved fruits, wild plants, and ornamental plants. It is notable for its delightful flowers, pleasant flavor, and delectable fruit.^7^ Peach is an important fruit crop of great economic importance, consisting of four botanical varieties: *P. persica* var. *nectarina* (Ait.) Maxim, *P. persica* var. *compressa* (Lond.) Bean, *P. persica* var. *densa Makino*, and *P. persica* var. *duplex* Rehd.^3^ Furthermore, it is closely related to five wild species, including *P. mira* (Koehne), *P. davidiana* (Carr.) Franch., *P. davidiana* var. potaninii (Batalin) Rehd., *P. kansuensis* Rehd., and *P. ferganensis* (Kost. and Rjab.)^4^. Peach (P. persica) varieties are classified into freestone and clingstone types based on how the flesh adheres to the pit.. Freestone peaches have flesh that naturally detaches from the stone, making them preferred for direct eating, while Clingstone peaches have flesh that clings tightly to the pit, which is preferred for canning due to its firmer texture. These textural differences are genetically controlled.^8^ Wild relatives represent an important genetic reservoir that enhances our understanding of plastome evolution and diversity in Prunus, which may provide a foundation for genetic resource utilization and future crop improvement efforts.^9^ Peach is a diploid species (2n = 2x = 16) with a relatively small genome size of approximately 230 Mbp.^10^, ^11^ Its small genome size makes it an ideal species for functional genomics research and a possible model plant for woody fruit species.^12^, ^13^ The plastome assembly of P. persica cv. Lovell^14^ marked an important step in advancing comparative plastome studies and in identifying DNA barcode genes in peach. Moreover, the decreasing cost of high-throughput plastome sequencing has created opportunities to generate additional cp genome sequences and to identify useful DNA barcodes from both coding and noncoding plastid genes in Prunus species.^15^ Most Prunus species have similar plastome lengths (∼158 kbp for the circular DNA molecule).^16^ The plastome generally harbors about 110–130 genes, including transfer RNAs (tRNAs), ribosomal RNAs (rRNAs), and protein-coding sequences, many of which are essential for photosynthesis.^4^, ^17^ The plastome is composed of a Large Single-Copy (LSC) region and a Small Single-Copy (SSC) region, separated by two Inverted Repeats (IRa and IRb). Its overall GC content generally ranges between 36% and 37%, with the IR regions displaying higher GC content than the LSC and SSC regions.^18^ Plastome sequences are highly valuable for resolving phylogenetic relationships, especially among closely related taxa, and for deepening insights into plant phylogeny and genome evolution. However, due to the high similarity among some Prunus taxa and potential complexities such as hybridization, species categorization can be challenging. The release of the complete peach nuclear genome^11^ together with the plastome of cv. Lovell,^14^ has significantly advanced comparative genomic studies and DNA barcode discovery in Prunus. However, understanding the evolutionary relationship between local and international cultivated varieties is critical for improving and selecting peach cultivars that can withstand forthcoming environmental changes. There is a need to generate more genetic information using Next-generation sequencing (NGS) to address the limited information on the genetic variation that controls local genotype adaptation to their surrounding environment.

The selection of the three peach materials, the cultivated Clingstone (CLS) and Freestone (FRS) types, and their wild relative *Prunus mira* (Koehne) was based on their distinct fruit stone adhesion types and contrasting domestication backgrounds. These characteristics make them suitable representatives for understanding plastome variation and evolutionary differentiation within Prunus. Therefore, this study aims to offer a comprehensive insight into plastome variation patterns by analyzing two edible peach cultivars along with a wild relative of *P. persica*. Through this research, we aim to compare the two peach specimens (*P. persica* cv. Clingstone [CLS] and Freestone [FRS]) with the wild relative *P. mira* (Koehne), and 22 other *Prunus* species from the NCBI database to explore the evolutionary relationships among these taxa. We also examine the DNA sequence diversity and evolutionary relationships of the three plastome genomes. The findings offer a comprehensive overview of peach plastome genome architecture, serving as a basis for developing molecular markers in clingstone (CLS) and freestone (FRS) peach varieties for phylogenetics, DNA barcoding, and biodiversity research.

## 2 Methods

### 2.1 DNA extraction and plastome genome sequencing

**Fig. 1:**
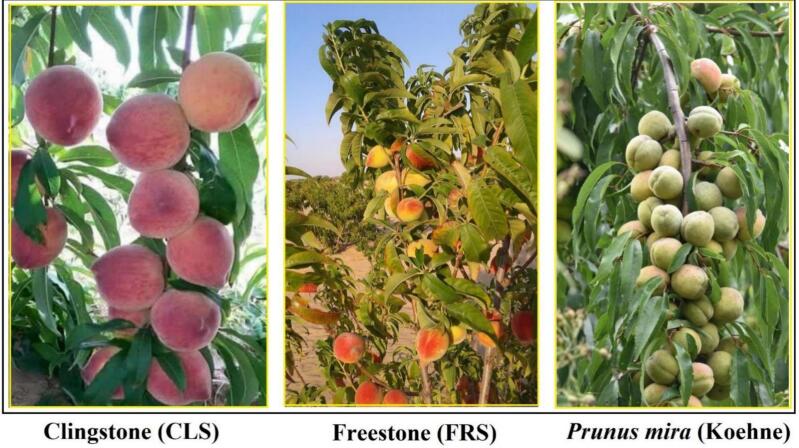
Fruit type variation among the three specimens; *Prunus mira* (Koehne), Freestone (FRS) and Clingstone cultivars (CLS, source: *https://www.courthousenews.com/fruits-of-prunus-mira/*).

This study utilized two peach cultivars, *P. persica* cv. Clingstone (CLS) and cv. Freestone (FRS), collected from Egypt, along with the wild relative *P. mira* (Koehne), which was sourced from the Wuhan Botanical Garden, Chinese Academy of Sciences (CAS), China. Fresh leaf tissue was collected from young trees during the spring growing season (Fig. 1). Genomic DNA was extracted using the Plant Genomic DNA Extraction Kit (DP305-03; Tiangen Biotech, Beijing, China) according to the manufacturer’s protocol. Genomic DNA was sequenced on the Illumina HiSeq 2500 platform. An Illumina plastome library was prepared using the DNA Library Prep Kit. Raw reads were filtered to remove low-quality regions and adapters, yielding high-quality clean data. The Geneious v. 10 tool was used to generate complete plastome genomes for each cultivar separately. The plastome assemblies were generated using the complete genome of *P. persica* cv. Lovell (GenBank accession HQ336405) as the reference.^14^

### 2.2 Structure comparison of peach plastome genomes

In this investigation, the three assembled plastomes were analyzed in comparison with 22 Prunus plastomes previously published in the NCBI GenBank database (Supplementary Table S1) in terms of genome content and structure. Three related species as outgroups: *Pyrus pyrifolia, Pyrus spinosa,* and *Malus prunifolia,* while plastome genome annotation was carried out using the Dual Organellar GenoMe Annotator (DOGMA) software ^19^. The genome of *P. persica* was used as the reference for annotation, and analyses of SNPs and InDels (insertion/deletion) were performed accordingly using MUMmer tool, where results were manually checked.^20^. MUMmer was used to identify and plot genomic regions showing potential inversions between all plastome genomes. IRSCOPE^21^ was used to assess and compare the inverted repeats and junction sites of plastome genomes across species. The genetic variation of gene structure and content was studied through multiple alignments of plastome genomes using Mauve^22^ and the comparative genomics tool of VISTA ^23^. OGDRAW (Draw Organelle Genome Maps) was used to draw the genome annotation of the studied plastome genomes.^24^

### 2.3 Simple Sequence Repeat analysis

The MISA tool was used to identify SSRs and compare them across the studied genomes, R scripts were applied.^25^The MegaSSR web server was used to analyze standalone microsatellites and to design targeted SSR PCR-based primers for the entire peach plastome.^26^ The MegaSSR tool was used to design PCR-based primers for selected SSR repeats and to initiate computational pipelines for SSR mining, classification, comparison, in silico PCR validation, and statistical visualization. A total of 14 genic and 19 non-genic SSR primers were investigated, as hotspot SSR regions, and selected as powerful SSR primers for the two cultivars *P. persica* cv. freestone (FRS) and *P. persica* cv. clingstone (CLS), respectively. All relevant information for SSR primers is presented in Supplementary Tables S2 and S3, including the cultivar name, sequence ID, process ID, repeat number, repeat type, repeat start, repeat end, primer start, primer end, repeat type, repeat length, gene, gene start, gene end, gene sequence strand, forward primer, forward Tm, forward size (bp), Forward GC, reverse primer, reverse Tm, reverse size (bp), reverse GC, product size (bp), and annotation.

### 2.4 The phylogeny studies

In the present study, 26 plastome genomes belonging to the family Rosaceae were used for the plastome phylogenetic relationships, including *P. persica* cv. Clingstone (CLS) and *P. persica* cv. freestone (FRS) specimens. According to a previous study,^27^ three species served as outgroups (*Pyrus pyrifolia, Pyrus spinosa,* and *Malus prunifolia*), and 46 protein-Coding Sequences (CDSs) were aligned in groups using MAFFT v.7.313 (supplementary Table S1).^28^ Gene arrangements were analyzed using MAFFT, while phylogenetic relationships among species were assessed through ML and BI models with IQ-TREE v.1.6.8 and MrBayes v.3.2.6, integrated in PhyloSuite v.1.2.2.^29^ ModelFinder was applied to determine the best-fitting model based on the Bayesian Information Criterion (BIC). For Bayesian analysis, the optimal model identified was JTT + F + R3, whereas for IQ-TREE it was GTR + R3 + F. Both models (JTT + F + R3 and GTR + R3 + F) were evaluated with 1000 bootstrap replicates using ultrafast bootstrapping^30^ and the Shimodaira–Hasegawa–like approximate likelihood ratio test.^31^ The phylogenetic tree obtained was ultimately visualized with FigTree v.1.4.2.^32^

### 2.5 Estimation of divergence time

Divergence dates were inferred using Bayesian analysis under a relaxed molecular clock to account for lineage-specific rate variation.^33^ Using BEAST v1.8.0, phylogeny and divergence times were estimated simultaneously under an uncorrelated relaxed lognormal model. Fossil calibrations assumed that the calibrated node (0) did not predate the earliest fossil occurrence, with normal priors set at a mean of 106.5, standard deviation of 1.0, and a 95 % upper bound corresponding to the stratigraphic age plus 10 %.^34^ For constraining the age of Prunus, the second node was assigned secondary calibration points using lognormal priors with an offset of 95, a mean of 0.5, and a standard deviation of 1.0.^35^ BEAUti v1.8.0 was used to create XML files for a partitioned dataset of 46 genes to estimate divergence times. An uncorrelated relaxed clock model was applied to infer molecular evolution rates and their variation, and the Yule speciation process was employed as the tree prior,^36^ the analysis started from a randomly generated tree, with uniform height priors assigned to Rosaceae ranging from 0 to 95 Mya, ensuring that these nodes did not predate the earliest known eudicot fossil evidence.^37^, ^38^ Prior constraints for node ages were assigned using a lognormal distribution, with the mean and standard deviation set according to the mean and median limits. The GTR + I + G model was applied as the nucleotide substitution model. Divergence time estimation was conducted using BEAST (v1.8.0) on XSEDE via the CIPRES web server. MCMC analyses were run for 10 million generations, sampling every 1,000 generations. Convergence and effective sample size (ESS > 200) were assessed using Tracer v1.7.1. Upon meeting these criteria, runs were combined with LogCombiner v1.8.0 after discarding the first 25 % of generations as burn-in. The resulting summary was generated in TreeAnnotator v1.8.0 to produce a maximum clade credibility tree with median node ages. Finally, the tree was visualized in FigTree v1.4.4 to display mean divergence times along with 95 % highest posterior density (HPD) intervals.

## 3 Results

### 3.1 Genome structural variation

**Fig. 2:**
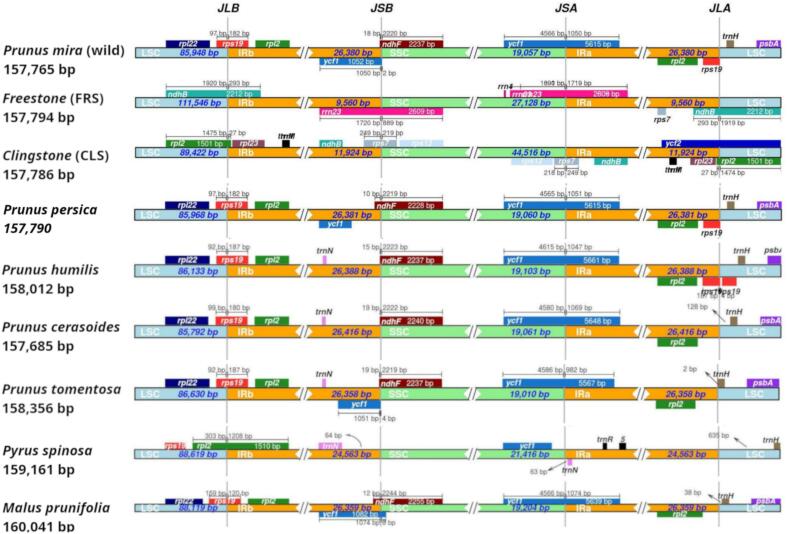
Comparison of quadripartite junction sites in *Prunus persica* plastome genomes. The start and end of each gene from the junctions have been shown with arrows. The T scale bar above or below the track shows genes integrated from one region of the plastome to another. JLB (IRb /LSC), JSA (SSC/IRa), JSB (IRb/SSC), and JLA (IRa/LSC) denote the junction sites between the quadripartite regions of the genome.

We sequenced the entire plastome genome of three different peach species. A genome-wide comparison was performed to investigate the genetic content and variations of 22 representative peach species and three outliers plastome plant genomes (*Pyrus pyrifolia, Pyrus spinosa* and *Malus prunifolia*) for which complete sequenced plastome were available (supplementary Table S1). Peach plastome genome size, structure, gene content, and other aspects of genome content were investigated. The genome sequence lengths of the sequenced peach plastome were 157,786 bp, 157,794 bp and 157,765 bp for two *P. persica* cv. clingstone (CLS) and freestone (FRS), and the wild type of *P. mira* (Koehne), respectively. The total number of genes in the plastomes ranged from 134 to 135. Most of the previously sequenced peach genomes show a similar genome length ranged from 157,685 bp (*Prunus cerasoides*) to 158,955 bp (*Prunus padus*) and a gene count ranges from 134 to 135 genes (supplementary Table S1 and Fig. 2).The peach plastome displays the typical genome organization observed in flowering plants. The A, T, G, C, and GC content in all plastome genomes were approximately 31.2 %, 32.1 %, 18 %, 19 %, and 37 %, with minor differences (supplementary Table S1).

### 3.2 Comparative analysis of IR contraction

To further explore the genomic differences contributing to size variation, the contraction and expansion of the Inverted Repeat (IR) regions were examined across all tested Prunus plastomes. At the LSC/IRs/SSC junctions, the contraction and expansion of inverted repeats were varied. The two IR regions divide the genome into two sections: large single copy (LSC) and small single copy (SSC). This may indicate that most *Prunus* species have a standard structural content of plastome genomes.

The average length of an IR region was 27,576 bp, except for *P. persica* cv. freestone (FRS) (9,560 bp), *P. persica* cv. clingstone (CLS) (11,924 bp), and *Pyrus spinosa* (24,563 bp). In the SSC region, the average length was approximately 20 Kbp, while *P. persica* cv. CLS and FRS exhibited SSC lengths of 27 Kbp and 44.5 Kbp, respectively. While the average size of the LSC region was 90,237 bp, with the exception of *P. persica* cultivar freestone (FRS), where the LSC exceeds the 100 kbp limit (11,1546 bp) (supplementary Table S1 and Fig. 2)**.**These results demonstrated considerable variation in the sizes of all six primary regions of the plastome genomes in *P. persica* (supplementary Table S1).

A detailed comparison of the four borders across the two IRs and two Single-Copy regions (SSC & LSC) revealed that the border structures across all peach species were highly similar (supplementary Fig. S1). *P. mira* (Koehne) exhibits a plastome structure similar to most Prunus species, except for the *ycf1* region in the IRb region, which is missing in roughly half of the studied taxa, including *P. persica* cv*.* clingstone (CLS) and freestone (FRS)*.* Moreover, one gene was absent in FRS and CLS (*ndh*B), and also two genes named *rrn*23 and *rpl*23 were absent in FRS and CLS, respectively (supplementary Fig. S1).

A slight difference in junction positions was found across the twenty-five plastome genomes studied. For instance, the length and location of the *rps 1*9 gene were variable across the IRa/LSC regions. In 25 plastome genomes, the *rps*19 gene was positioned at the LSC/IRb junction, whereas in three specimens *P. persica* cv. FRS, CLS, and *P. spinosa* resided within the IRb region, with lengths ranging from 62 to 240 bp.

Additionally, a crossover was observed in *P. persica* cv. in CLS and *P. spinosa.* The *rps*19 gene is located at the LSC/IRb junction, whereas the *rpl22* gene is entirely contained within the IRb region in all examined species (supplementary Fig. S1)**.** In all species, the *ndhF* gene was located in the SSC region, except in four specimens *P. persica* cv. FRS, CLS, *P. serotina*, and *P. spinosa*. Minor crossover variations of 4–19 bp were observed at the IRb/SSC junction of the *ndhF* gene (supplementary Fig. S1). These junctional variations revealed a unique rearrangement of several gene regions, leaving a distinct footprint for the evolutionary lineage through which it passed.

**Fig. 3:**
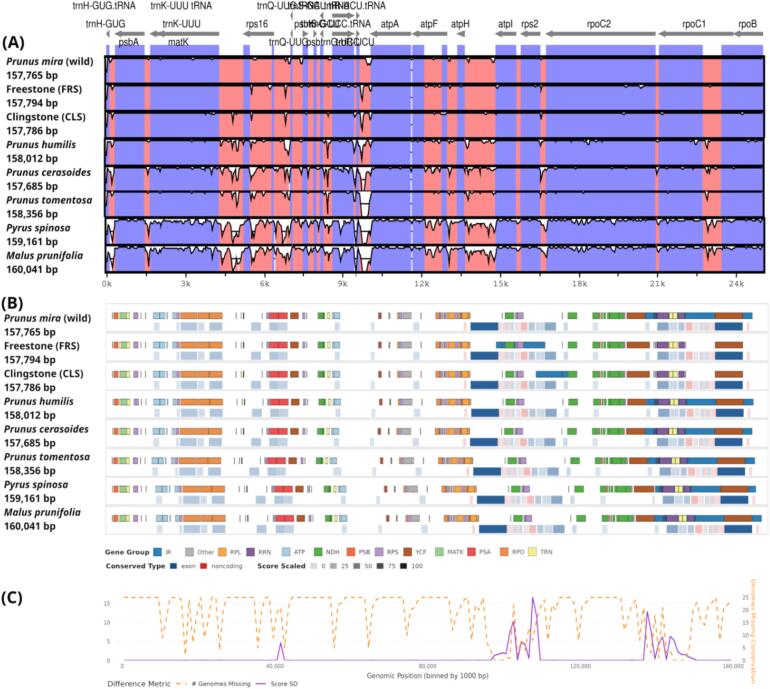
Sequence alignment and comparative analysis of plastome genomes from selected Prunus species among the 25 studied taxa. The top panel shows a VISTA-based identity plot using *Prunus persica* as reference, with regions color-coded as protein-coding (blue), rRNA (purple), tRNA (green), and conserved non-coding (pink). The y-axis indicates sequence identity (50–100 %), with dips marking divergent hotspots. The middle panel summarizes conserved genomic intervals and differences across species; gene blocks are colored by function, and gray regions indicate inverted repeats (IRs). The bottom panel displays genome-wide variation metrics: orange dashed lines show the number of genomes missing per 1,000 bp bin, and the purple line represents standard deviation in conservation scores. (For interpretation of the references to color in this figure legend, the reader is referred to the web version of this article.)

The plastome genome of *P. persica* was compared with those of the other accessions using dot-plot analyses. The results revealed that, except for *P. persica*, the remaining accessions retrieved from NCBI showed no major rearrangements and were fully collinear, with only a few small insertions and deletions observed (Fig. 3). The sequence alignment of plastome genomes has been conducted using different bioinformatics tools (supplementary Fig. S2). We detected several genomic regions that inferred some genomic variations, although the sequence similarity indicates low variation rate, for instance, (supplementary Fig. S2) shows that *ycf2* has some sequence length variations across different species.

Unique gene rearrangements as well as IR contraction and expansion were further examined using Mauve alignment. The plastome genomes of all other species exhibited high similarity, forming largely collinear blocks, with the exception of *P. dulcis* and *P. serotina*. Variations in these two genomes, including IR contraction and expansion, changes in gene content, and gene rearrangements, were further analyzed and visualized through the collinear blocks generated by the Mauve alignment (supplementary Fig. S3). The sequence alignment statistics indicated that the alignment length was 168,300 bp, where the number of variable sites was 0.053 %, and the parsimony informative sites were 0.037 %.

### 3.3 Simple Sequence Repeats and gene variation

Simple sequence repeat (SSR) loci are among the most variable regions in the plastome, and their distribution patterns offer important insights into the genome evolution and genetic differentiation of *Prunus* taxa. An evaluation of SSR motifs across 25 plastome sequences revealed notable diversity in *Prunus* and *Malus* species. This study identified numerous polymorphic SSR loci, including mono (p1), di (p2), penta (c), and *hexa*-nucleotide (c*) repeats, respectively. Whereas tri and tetra-nucleotide SSR repeat were absent in all specimens tested. On average, the highest total SSRs number discovered in the 25 plastome genome species was as follows: 1064 (*P. serrulata*), 1035 (*P. cerasoides*) and 980 (*P. pseudocerasus*), while the lowest total SSRs number being 671 (*P. mume*), 620 (*P. yedoensis*) and 595 (*P. mira* Koehne) (supplementary Table S1). On the other hand, a total of 807, 775 and 559 SSRs numbers were observed in the two tested specimens in this study *P. persica* cv. FRS, CLS and the wild relative *P. mira* (Koehne), respectively. In detail, within the Prunus species, the type (p1) repeat is the most common among the 25 species, followed by type (c) and type (p2), respectively. Additionally, no type (c*) repeat was present in 15 species out of 25 specimens tested as shown in (supplementary Table S1 and supplementary Fig. S4).

Furthermore, the MegaSSR web server was utilized for the large-scale identification, classification, and development of SSR markers. This platform provides a comprehensive suite of tools that automate the identification, classification, and annotation of SSRs. MegaSSR generates multiple visual representations to summarize the results, as shown in Supplementary Figs. S5–S11. These visualizations include, common repeats shared between genic and non-genic regions, distribution and frequency of different SSR classes and motifs, SSR distribution, accounting for sequence complementarity, unique repeats specific to genic and non-genic regions, and a venn diagram illustrating the overlap of SSRs between genic and non-genic regions.

The underlying data for these analyses were derived from the coordinates of specific SSR units or PCR primer target regions. Collectively, the findings demonstrate significant interspecific variation in SSR patterns. This variation provides crucial insights for future studies using SSR molecular markers and supports the development of DNA barcoding methods in the genus *Prunus*.

### 3.4 Codon usage analysis

**Fig. 4:**
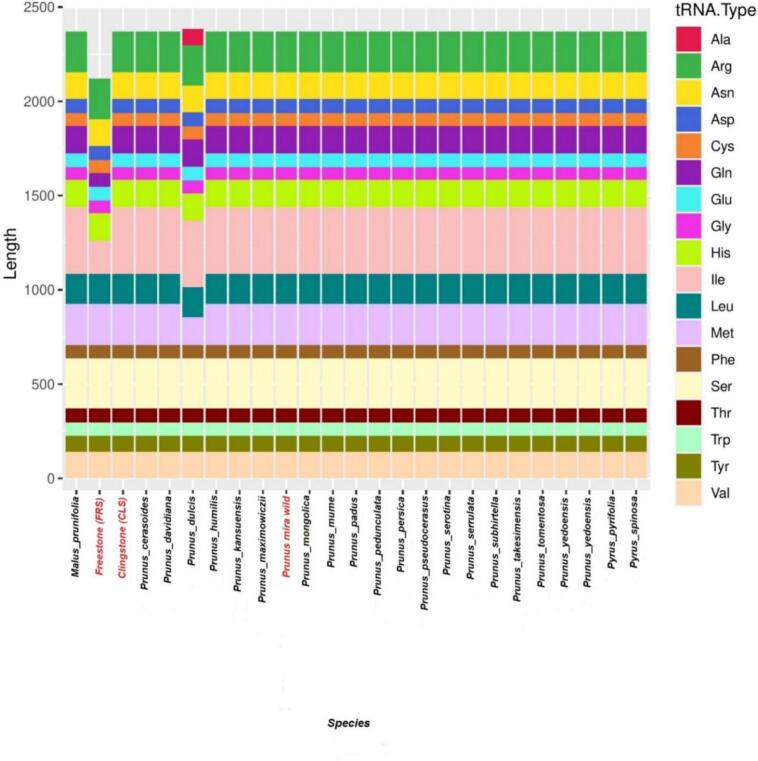
Amino acid frequencies of the *Prunus persica* protein-coding sequence.

**Fig. 5:**
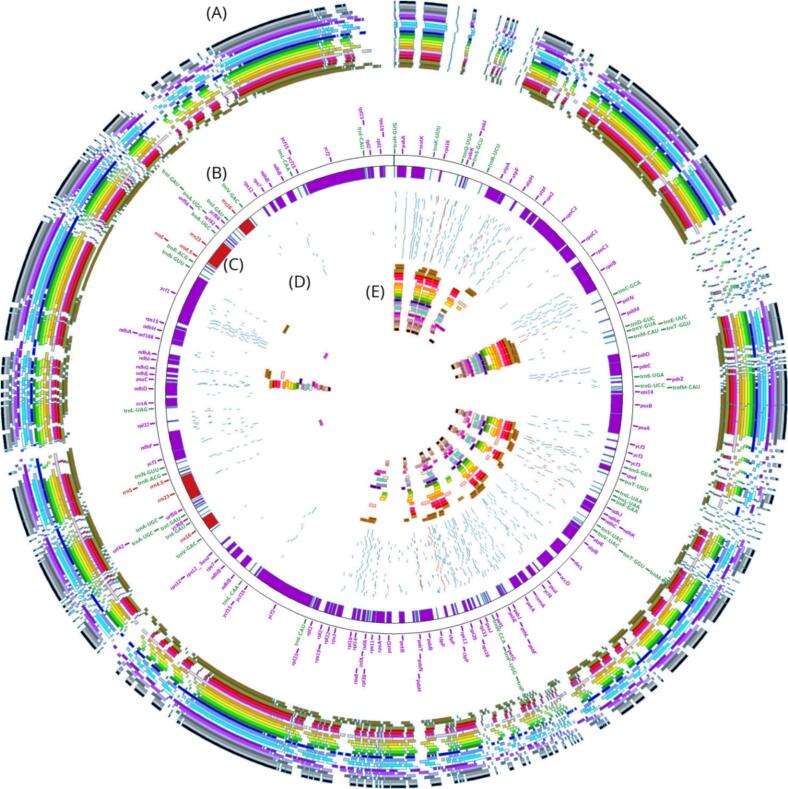
Overview of plastome variation in *Prunus persica*. (A) The outermost ring shows the multiple sequence alignment of complete plastome sequences from the analyzed accessions, illustrating conserved regions and alignment gaps. (B) Gene annotation track displaying plastid gene organization, including protein-coding genes (purple), tRNA genes (green), and rRNA genes (red), with gene names shown along the circumference. (C) Highlighted plastid gene locations across the plastome, representing the physical distribution of annotated genes. (D) Distribution of simple sequence repeat (SSR) loci across the plastome, including different repeat types indicated by distinct colors. (E) Comparative plastome similarity based on BLAST analysis, illustrating sequence conservation and divergence among the studied plastomes. (For interpretation of the references to color in this figure legend, the reader is referred to the web version of this article.)

To complement the analysis of SSR variability, we examined codon usage to assess differences in gene expression and amino acid preferences. This analysis reveals the evolutionary pressures on coding sequences and provides further insight into the functional conservation or divergence of plastid genes among *Prunus* species. The tRNA genes carry the information to synthesize all 20 amino acids necessary for protein production. With the exception of Peach cv. clingstone (CLS, 2,120 bp), among the twenty-four accessions, the amino acid frequencies in plastome protein-coding sequences showed variation, with isoleucine and glutamine represented by 177 bp and 71 bp, respectively in *P. persica* cv. FRS (Table 1). Moreover, the findings indicate that tRNA in the *P. persica* plastome structure may transport 20 amino acids for protein production. Because tRNA types and lengths differ throughout species, the distribution of tRNA lengths across Prunus and Malus species varies (Fig. 4) and highlights genetic variation. *P. dulcis* and *P. persica* cv. FRS cultivars, in particular, have notably large tRNA lengths for alanine (Ala) and glycine (Gly). *P. mira* (Koehne) and *P. pseudocerasus* lose alanine, while *P. kansuensis* and *P. humilis* both lose glycine. Thinning of the Pyrus spinosa tRNA and lengthening of the arginine (Arg) tRNA were observed in Malus prunifolia. The assembled accessions of *P. persica* were found to have a substantially conserved number of genes, 40 total genes, 28 single-copy and 12 multi-copy genes were found by gene annotation in all constructed plastome genomes (Fig. 5), which illustrates gene distribution, GC content, and sequence divergence among the Clingstone, Freestone, and *P. mira* plastomes, providing a clear visualization of conserved and variable genomic regions within *Prunus*. These comprised six protein-coding genes, sixteen transfer RNA (tRNA) genes, and four ribosomal RNA (rRNA) genes. Seven annotated genes among all species were found to have introns: two in *ycf3*, a single copy was identified in *rps*12, with one copy each in *trnK-UUU*, *trnG-UCC*, *trnL-UAA, trnI-GAU*, and *trnA-UGC*. The IR region contained four rRNA genes (*rrn16, rrn23, rrn4.5*, and *rrn5*), four tRNA genes (*trnI-CAU*, *trnL-CAA, trnV-GAC,* and *trnA-UGC*), and three protein-coding genes (*ycf2, ycf1,* and *ndhB*). The SSC region included one tRNA gene (*trnN-GUU*) and two protein-coding genes (*ndhF* and *ndhD*). With the exception of *rps12*, the LSC region included 18 genes total, comprising 3 protein-coding genes (*atpB*, *rbcL*, and *accD*), and 15 tRNA genes were also identified. Of particular interest, *rps*12 contained two *trans*-splicing introns, with one exon located in the LSC region and the other duplicated in the IRs (Fig. 5). Furthermore, in the plastome genomes, *matK, rbcL, psaA, psbA, psbC, rps16, rpl2, rpl16, ndhA, ndhD, ndhF,* and *ndhH* genes were found to be associated with SSRs.

**Table 1:** Summary of the complete plastome genome characteristics of three *Prunus* species.

Species	Genome size (bp)	LSC size (bp)	SSC size (bp)	IR size (bp)	Number of genes	Protein coding genes	tRNA genes	rRNA genes	GC%
*Prunus mira (Koehne)*	157.765	85.948.	19.057	26.380	135	94	36	5	36.8
*Freestone* (FRS)	157.794	111.546	27.128	9.560	134	94	36	5	36.8
*Clingstone*(CLS)	157.786	89.422	44.516	11.924	135	94	36	5	36.8

### 3.5 Plastome phylogenomic analysis

**Fig. 6:**
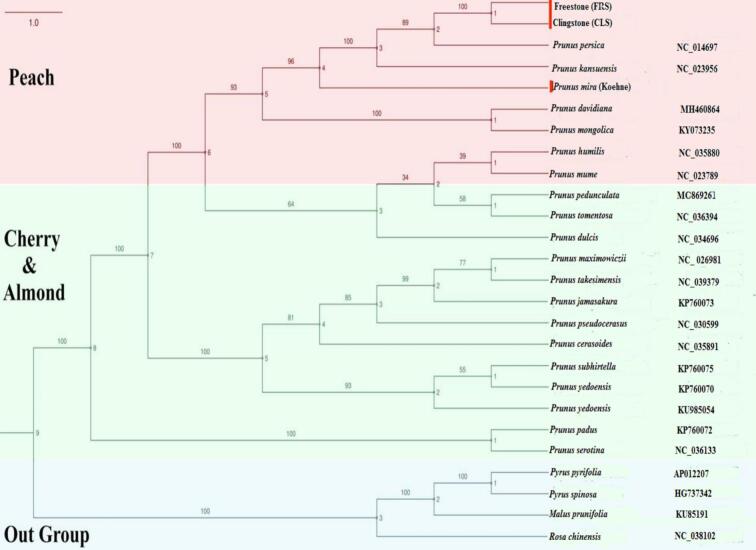
Phylogenetic tree based on whole plastome genome sequences of Prunus species and other selected taxa used as references, outgroups, or for comparative purposes. Numbers on the branches represent bootstrap support values, and GenBank accession numbers are provided. Plastomes of peach isolates generated in this study are highlighted in red. (For interpretation of the references to color in this figure legend, the reader is referred to the web version of this article.)

To further investigate the genetic relationships and evolutionary history, we reconstructed a phylogeny of the studied Prunus species based on the identified structural and sequence variations. This analysis aimed to clarify the placement of the Clingstone and Freestone cultivars relative to their wild relative, *P. mira*, and other *Prunus* taxa, providing a broader perspective on lineage divergence within the genus. A maximum likelihood (ML) phylogenetic tree was constructed, using three *Pyrus* and *Malus* species as outgroups (Fig. 6). The resulting tree robustly divided the 23 Prunus specimens into three major clades with strong bootstrap support, corresponding to the peach, almond, and cherry groups, thereby providing a broader perspective on lineage divergence within the genus. In detail, the two peach varieties, *P. persica* cv. FRS and CLS, clustered with *P. persica* cv. Lovell to form a monophyletic clade, which was further associated with *P. kansuensis* and *P. mira* (Koehne). *P. davidiana, P. mongolica, P. humilis,* and *P. mume* were positioned at the base of this clade, indicating their close genetic relationship with peach. Additionally, all almond and cherry species grouped together in a second monophyletic clade, showing a close evolutionary relationship with the peach clade. This clade was further subdivided into four distinct monophyletic groups representing the almond and cherry lineages. This indicates considerable genetic diversity within cherry and almond species. A unifying clade (clade two) included 14 members of cherry and almond, namely *Prunus pedunculata*, *Prunus tomentosa*, *Prunus dulcis*, *Prunus maximowiczii*, *Prunus takesimensis*, *Prunus jamasakura*, *Prunus pseudocerasus*, *Prunus cerasoides*, *Prunus subhirtella*, *Prunus yedoensis*, *Prunus padus*, and *Prunus serotina*. In contrast, a black cherry (*Prunus rosa* var. spontanea) occupied the basal position of the cherry clade independently. Meanwhile, *Pyrus pyrifolia, Pyrus spinosa*, and *Malus prunifolia* were positioned individually as outgroups of the tree.

### 3.6 Divergence time estimation analysis

**Fig. 7:**
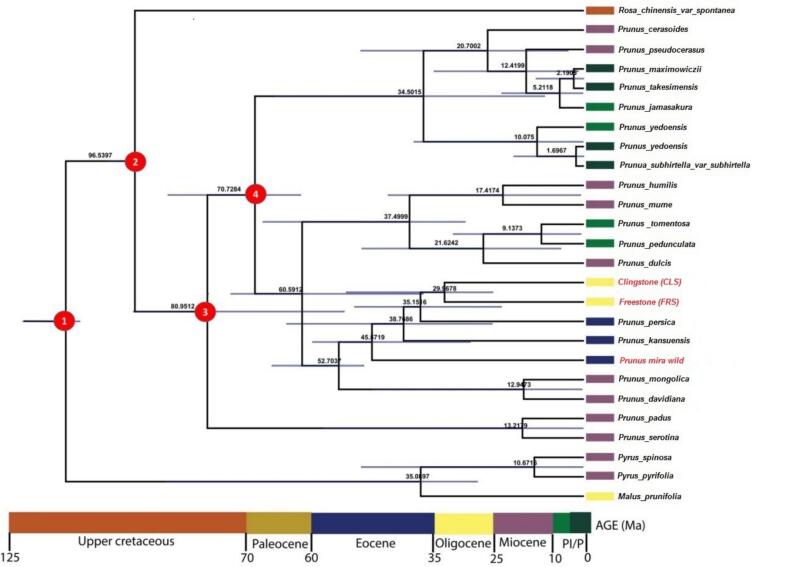
Phylogenetic chronogram showing the evolutionary dating time of order *Prunus* using 26 taxa. The tree was estimated using Bayesian analysis of 46 protein-coding genes in the MCMC tree. The number in the circle in red relates to our two nods of interest. (For interpretation of the references to color in this figure legend, the reader is referred to the web version of this article.)

To place these phylogenetic relationships in an evolutionary context, we estimated divergence times using fossil calibrations and a molecular clock analysis. This approach provided a temporal framework for Prunus diversification, estimating the timing of major speciation events and revealing when the Clingstone, Freestone, and *P. mira* lineages diverged from their common ancestors. The estimated root age of Prunus (node 1) was approximately 96.54 million years ago (Ma) (95 % HPD: 125–70 Ma), placing its origin in the Upper Cretaceous period (Fig. 7). The divergence of the genus Prunus (node 2) occurred around 80.95 Ma (95 % HPD: 125–70 Ma), also within the Upper Cretaceous. P. serotina (node 3) diverged at about 70.73 Ma (95 % HPD: 70–60 Ma), corresponding to the Paleocene. The split between P. cerasoides and P. pseudocerasus (node 4) was estimated at 60.59 Ma (95 % HPD: 60–50 Ma), during the Paleocene. P. persica diverged later, around 35.15 Ma (95 % HPD: 36–25 Ma) in the Oligocene, and subsequently differentiated into various cultivars approximately 10.67 Ma (95 % HPD: 11–1 Ma) in the Miocene. The most recent diversification among P. persica cultivars occurred near 0.476 Ma.

## 4 Discussion

Assessing genetic variation between wild and cultivated species is key to incorporating important traits from wild relatives into cultivated varieties. In this study, with the swift progress in next-generation sequencing (NGS) technologies, we assembled the plastome genomes of two edible *P. persica* cultivars clingstone (CLS) and freestone (FRS) in addition to the wild relative *P. mira* (Koehne) and were compared to previously published peach genomes.

Several studies have recently published plastome genome sequences obtained by whole genome sequencing.^39^ Most Prunus species have a similar plastome genome length with numerous genomic regions that exhibit some genomic variations, although the sequence similarity indicates a low variation rate within the CDS. This variation results from a decreased substitution rate within the genus.^27^ Previous evidence in Rosaceae suggests that,^40^ while the plastome genome remains largely conserved in terms of structure and linear gene order, Prunus species display notable differences in gene content and arrangement.^4^

In our analysis, the plastome genomes of *P. persica* cv. freestone (FRS), *P. persica* cv. clingstone (CLS), and the wild relative *P. mira* (Koehne) were similar in size, measuring 157,794 bp, 157,786 bp, and 157,765 bp, respectively, and encoded 130–133 genes. The results revealed only minor differences in GC content and composition, indicating that the overall plastome structure is relatively stable. Nevertheless, some highly variable regions were identified, while the overall evolutionary rate remains low (Fig. 3). Most of these variations were found in non-coding regions, which largely contribute to differences in plastome genome size. These regions also exhibit higher levels of sequence diversity, making them useful for developing DNA barcodes to assess phylogenetic relationships at the subspecies level.^6^, ^41^

In *Prunus persica*, the plastome genome exhibits a circular structure composed of an LSC, an SSC, and two IR regions. Variations in genome size among plant lineages are often associated with IR expansion and contraction, which can provide insights into phylogenetic relationships and genome evolution.^42^ Three possible explanations for the diversity of the IR border region sequences are intermolecular recombination, the presence of numerous repeat sequences and InDels that led to mismatches, resulting in the upstream sequence being converted into a single copy^43^. In agreement with Kim and Lee,^44^ these findings suggest that expansion and contraction of the IR and single-copy (SC) boundary regions are the primary mechanisms driving length variation in angiosperm plastome genomes.

Additionally, IR regions showed lower sequence divergence compared to the LSC and SSC regions, a pattern commonly observed in higher plants, likely due to copy correction between IR sequences via gene conversion.^45^ Given that the content of unique genes is conserved across all species, our results further support the highly conserved nature of peach plastome genomes. A similar pattern of IR expansion has been observed in the plastome genome of *Prinsepia utilis* Hayata, also belonging to the Rosaceae family.^46^ However, in Prunus, evidence from previous studies indicates that the higher levels of variation observed in the IR, LSC, and SSC regions—relative to previously sequenced plastome genomes—may represent hotspots of genetic diversity, thereby facilitating the discrimination of these regions from more conserved portions of the genome.^15^

At lower taxonomic levels, highly variable coding plastid DNA, such as the two-locus *ycf1–ndhF* region, serves as an effective and promising DNA barcode in Prunus. The elevated variation observed in the IR, LSC, and SSC regions compared to previously sequenced plastomes may indicate hotspots of genetic diversity, distinguishing these regions from other parts of the genome. This idea has gained substantial support from recent plastome genome studies.^27^, ^47^

Simple sequence repeats are important driving factors that control whole genome size and evolution; additionally, developing repeatable and stable SSR markers may aid future genetic research in peach. Previous studies have suggested that repeated sequences may play a vital role in sequence rearrangements of complete plastome genomes.^48^ Herein, numerous polymorphic SSR loci have been identified in the 25 plastome genomes ranging from 1064 to 559 units including mononucleotide, dinucleotide, pentanucleotide and hexanucleotide sequences, mono-nucleotide repeats were the most abundant among Prunus species, consistent with previous reports in flowering plants.^48^ Evidence suggests that divergence has occurred in the majority of SSRs between wild and cultivated peaches.^49^, ^50^, ^51^ These genic SSR repeat sequences comprise some highly diverged hotspot regions with different rates of mutational events and thus provide candidate molecular markers These regions may serve as suitable markers for DNA barcoding, phylogenetic analysis, and molecular evolution studies within the Rosaceae family at lower taxonomic levels.

Codon usage, common across many plant species, describes the unequal frequency of synonymous codons in the genome, reflecting a fundamental and complex aspect of genetic coding.^52^ Recently, codon usage has been closely studied in *Prunus*, gene families, and the whole genome.^53^, ^54^ Because tRNA types and lengths differ throughout species, the distribution of tRNA lengths across *Prunus* and *Malus* species differs, highlighting genetic variation. Herein, we examined the codon usage patterns and their determinants in the protein-coding genes of *P. persica* plastome genomes, which display a conserved gene structure. Across all assembled plastomes, gene annotation revealed 40 genes, including 28 single-copy and 12 multi-copy genes. Our findings suggest that the tRNA in the *P. persica* cv. FRS cultivars and *P. dulcis* has particularly large tRNA lengths for alanine (Ala) and glycine (Gly), which is likely involved in transporting 20 amino acids necessary for protein production, consistent with research on other angiosperm plastome genomes.^55^ Although variations exist, codon usage provides valuable information on gene expression and evolutionary adaptations, and could serve as an effective genomic marker for DNA barcoding and evolutionary analyses within the Rosaceae family.

Prunus is among the largest genera in the Rosaceae family, characterized by a complex evolutionary history involving high genetic diversity, frequent hybridization, and intricate morphological traits, all of which complicate phylogenetic studies. Plastome phylogenomics analyses have clarified longstanding ambiguities in the classification and evolutionary history of complex plant families like Rosaceae, offering insights that were previously difficult to achieve using traditional phylogenetic methods.^56^ As shown in our plastome phylogenomic tree, the two peach varieties, *P. persica* cv. clingstone (CLS) and freestone (FRS), clustered with *P. persica* cv. Lovell to form a monophyletic clade, which further grouped with *P. kansuensis* and *P. mira* (Koehne), distinct from other wild relatives. This pattern supports previous observations of plastome genome introgression among Prunus species, consistent with traditional classifications.^5^, ^57^ Evidence suggests that *P. mira* (Koehne) represents the oldest ancestor of peach and is regarded as a traditional wild germplasm for peach breeding. Our results also indicate a close relationship between *P. davidiana* and *P. mongolica*, while *P. kansuensis* appears more closely related to *P. persica*, in agreement with previous genome re-sequencing studies.^3^, ^58^ Furthermore, *P. mira* (Koehne) was found to be closely related to *P. mongolica*, *P. davidiana*, and *P. dulcis*, indicating a potential common ancestry and supporting the proposed hybrid origin of peach involving almond, in agreement with earlier studies.^53^ Similarly, *P. mume* is more closely related to the three cherry species *P. humilis*, *P. pedunculata*, and *P. tomentosa*, yet diverges from the other cherry species, suggesting that plastome genome introgression from plum to cherry may have occurred.^4^, ^5^ Furthermore, our phylogenomic analyses provide strong support for the monophyly of *P. pseudocerasus*, highlighting its status as the rootstock for Chinese cherry species.^35^ These results align with previous studies, indicating that domestication events led to the separation of cultivated peach from wild relatives and cherry species.^59^

Estimated divergence times for the 26 species indicate a shared common ancestor around 96.54 Mya, with the two primary clades separating approximately 10.67 Mya (95 % HPD: 70.7–80.9 Mya). The fossil evidence placing the origin of *P. persica* in the late Pliocene highlights the deep evolutionary roots of the peach species. The discovery of this fossil has provided valuable insights into the evolutionary history of peaches, pushing back the estimated timeline of domestication and diversification, as demonstrated in previous studies.^60^ Our results indicate that *P. persica* diverged from the peach cultivars *Prunus persica* cv. clingstone (CLS) and freestone (FRS) approximately 6 Mya. Furthermore, the estimated evolutionary patterns within Rosaceae suggest that hybridization and polyploidy may have played a crucial role in the family’s early evolution during the Eocene.^48^, ^61^ Previous evidence suggests that the Rosaceae diverged during the Eocene (53–36.5 Mya), consistent with the results of our study.^29^

In summary, our study demonstrates that plastomes provide valuable insights for resolving relationships among closely related species and subgenera, tracing maternal lineages, and understanding the domestication history of peach. Our results offer a reference for hotspot regions that will be valuable for future studies, especially for differentiating and identifying Clingstone and Freestone cultivars in peach evolution.

## 5 Conclusion

This study provides a comprehensive comparative analysis of plastome genomes of *Prunus persica* cultivars (Clingstone and Freestone) and their wild relative *P. mira* (Koehne), revealing variations that contribute to phylogenetic understanding within the genus Prunus, together with 22 additional Prunus species, enhancing the available plastome genomic resources for subgenus Prunus. All 25 specimens displayed the typical quadripartite plastome structure, with genome sizes ranging from 157,685 bp in *P. cerasoides* to 158,955 bp in *P. padus*, and an average GC content of 37.72 %. Our findings highlighted significant structural variations, particularly at the IR/LSC/SSC junctions, as well as distinct gene rearrangements across Prunus species. Highly variable regions, such as SSR repeats and codon usage patterns, represent potential markers for DNA barcoding and evolutionary studies, although their full potential remains to be established. These divergence-prone regions largely account for plastome genome size variation, especially at lower taxonomic levels. Phylogenomic analyses further support well-defined monophyletic clusters, providing clear separation among peach, cherry, and almond species without overlap. Divergence time estimates offered further insights into the evolutionary history of Prunus, suggesting that the clingstone (CLS) and freestone (FRS) peach cultivars, along with the wild relative *P. mira* (Koehne), diverged around the Oligocene period, approximately 35.15 Mya. Comparative analyses of plastome structural variations provide novel perspectives on the evolution of these peach cultivars, with potential applications for DNA barcoding to assess interspecific variation at the subspecies level in *P. persica*, which will aid future phylogeny and species identification.

## 6 Funding Declaration

This study did not receive any funding.

## 7 Data Availability

All raw sequencing data have been deposited in the NCBI database under the following submission numbers: PX764274 (Freestone FRS), PX764275 (Clingstone CLS), PX764276 (*Prunus mira)*. The corresponding genomic FASTA files and GenBank annotation files are provided as supplementary materials with this article. Additionally, data and computational codes used in this article can be found at this online resource: https://doi.org/10.5281/zenodo.15687829.

## CRediT authorship contribution statement

**Mokhtar Said Rizk:** Writing – review & editing, Writing – original draft, Visualization, Methodology, Formal analysis, Conceptualization. **M. Alsamman:** Writing – review & editing, Writing – original draft, Software, Methodology, Conceptualization. **Heba A. M. AbdAlla:** Writing – review & editing, Writing – original draft, Visualization, Software, Methodology, Formal analysis. **Mohamed Abd.S. El Zayat:** Writing – review & editing, Writing – original draft, Visualization, Supervision, Resources, Methodology. **Ahmed H. Hassan:** Writing – review & editing, Writing – original draft, Methodology, Funding acquisition. **El-Shaimaa Saad El-Demerdash:** Writing – review & editing, Writing – original draft, Visualization, Supervision, Investigation, Formal analysis, Data curation. **Mohamed Z. S. Ahmed:** Writing – review & editing, Writing – original draft, Visualization, Software, Data curation, Conceptualization. **Yuepeng Han:** Writing – review & editing, Writing – original draft, Supervision. **Mohamed Hamdy Amar:** Writing – review & editing, Writing – original draft, Supervision, Formal analysis, Data curation, Conceptualization. **Achraf El Allali:** Writing – review & editing, Writing – original draft, Formal analysis.

## Conflict of interest

The authors have declared that no competing interests exist.
