# Supplementary material for: Structural analysis of clingstone and freestone peach (Prunus persica L.) plastome genome: provides insight into phylogeny and time diversification
Source: J Genet Eng Biotechnol. 2026 Feb 28;24(1):100660. doi: 10.1016/j.jgeb.2026.100660 (PMC12966720; doi:10.1016/j.jgeb.2026.100660)
Supplement: Supplementary Data 1 [file mmc1.docx]

Supplementary Tables

**Table 1.** Summary of plastome genomes used for studying peach genomic accessions.

| Species  **9/19** | Accession | Study | Len  .(bp) | GC  (%): | A  (%) | T  (%) | G  (%) | C  (%) | Genes | IRs  (bp) | SSC  (bp) | LSC  (bp) | SSR | | | | | |
| --- | --- | --- | --- | --- | --- | --- | --- | --- | --- | --- | --- | --- | --- | --- | --- | --- | --- | --- |
|  |  |  |  |  |  |  |  |  |  |  |  |  | No. | p1  (bp) | p2  (bp) | C  (bp) | c*  (bp) |  |
| *Prunus mira* koehne wild | - | This | 157765 | 36.74 | 31.1 | 32.1 | 17.98 | 18.76 | 135 | 26380 | 19057 | 85948 | 47 | 458 | 36 | 65 | - | 559 |
| Freestone (FRS) | - | This | 157794 | 36.71 | 31.08 | 32.1 | 17.96 | 18.75 | 134 | 9560 | 27128 | 111546 | 60 | 590 | 36 | 180 | - | 806 |
| Clingstone (CLS) | - | This | 157786 | 36.71 | 31.07 | 32.08 | 17.96 | 18.75 | 135 | 11924 | 44516 | 89422 | 57 | 559 | 36 | 180 | - | 775 |
| *Prunus mume* | KF765450.1 | - | 157712 | 36.74 | 31.11 | 32.13 | 17.99 | 18.75 | 135 | 26395 | 19062 | 85860 | 57 | 586 | 50 | 35 | - | 671 |
| *Prunus maximowiczii* | KP760071.1 | - | 157852 | 36.72 | 31.15 | 32.11 | 18 | 18.71 | 135 | 26436 | 19133 | 85847 | 63 | 569 | 12 | 319 | 30 | 930 |
| *Prunus serrulata* | KP760073.1 | - | 157882 | 36.72 | 31.16 | 32.11 | 18 | 18.72 | 135 | 26397 | 19120 | 85968 | 69 | 570 | 24 | 440 | 30 | 1064 |
| *Prunus subhirtella* | KP760075.1 | - | 157833 | 36.73 | 31.15 | 32.11 | 17.99 | 18.73 | 135 | 26381 | 19120 | 85951 | 60 | 547 | 24 | 272 | 25 | 868 |
| *Prunus yedoensis* | KU985054.1 | - | 157792 | 36.74 | 31.15 | 32.1 | 17.99 | 18.74 | 135 | 26379 | 19120 | 85914 | 47 | 478 | 24 | 93 | 25 | 620 |
| *Prunus mongolica* | KY073235.1 | - | 158451 | 36.72 | 31.15 | 32.12 | 17.97 | 18.74 | 135 | 26391 | 19144 | 86525 | 57 | 545 | 48 | 176 | - | 769 |
| *Prunus pedunculata* | MG869261.1 | 46 | 157873 | 36.78 | 31.11 | 32.1 | 17.99 | 18.78 | 135 | 26385 | 19029 | 86074 | 58 | 590 | 24 | 170 | - | 784 |
| *Prunus persica* | NC_014697.1 | 12 | 157790 | 36.75 | 31.11 | 32.12 | 17.98 | 18.77 | 135 | 26381 | 19060 | 85968 | 60 | 590 | 36 | 180 | - | 806 |
| *Prunus kansuensis* | NC_023956.1 | - | 157736 | 36.75 | 31.1 | 32.13 | 17.98 | 18.77 | 135 | 26387 | 19122 | 85840 | 55 | 529 | 62 | 153 | - | 744 |
| *Prunus yedoensis* | NC_026980.1 | - | 157859 | 36.73 | 31.15 | 32.11 | 17.99 | 18.73 | 135 | 26381 | 19120 | 85977 | 60 | 546 | 24 | 272 | 25 | 867 |
| *Prunus padus* | NC_026982.1 | - | 158955 | 36.59 | 31.24 | 32.16 | 17.91 | 18.67 | 134 | 26209 | 18871 | 87666 | 63 | 507 | 62 | 342 | - | 911 |
| *Prunus pseudocerasus* | NC_030599.1 | 47 | 157834 | 36.73 | 31.15 | 32.1 | 18 | 18.72 | 135 | 26393 | 19084 | 85964 | 64 | 552 | 24 | 376 | 28 | 980 |
| *Prunus dulcis* | NC_034696.1 | - | 157723 | 36.77 | 31.12 | 32.09 | 18 | 18.76 | 135 | 26401 | 18952 | 85969 | 52 | 490 | 62 | 147 | - | 699 |
| *Prunus humilis* | NC_035880.1 | 48 | 158012 | 36.61 | 31.09 | 32.07 | 17.91 | 18.7 | 135 | 26388 | 19103 | 86133 | 54 | 534 | 30 | 215 | - | 779 |
| *Prunus cerasoides* | NC_035891.1 | 50 | 157685 | 36.72 | 31.14 | 32.13 | 17.99 | 18.72 | 135 | 26416 | 19061 | 85792 | 63 | 581 | 12 | 420 | 22 | 1035 |
| *Prunus serotina* | NC_036133.1 | 51 | 158778 | 36.62 | 31.2 | 32.16 | 17.94 | 18.68 | 134 | 26294 | 18909 | 87281 | 70 | 675 | 26 | 239 | - | 940 |
| *Prunus tomentosa* | NC_036394.1 | 52 | 158356 | 36.85 | 31.06 | 32.07 | 18.02 | 18.83 | 135 | 26358 | 19010 | 86630 | 56 | 551 | 48 | 168 | - | 767 |
| *Prunus takesimensis* | NC_039379.1 | 53 | 157948 | 36.71 | 31.17 | 32.11 | 17.99 | 18.71 | 135 | 26436 | 19117 | 85959 | 64 | 544 | 24 | 375 | 29 | 972 |
| *Prunus davidiana* | NC_039735.1 | 54 | 158055 | 36.75 | 31.12 | 32.11 | 17.99 | 18.76 | 135 | 26380 | 19047 | 86248 | 53 | 510 | 48 | 171 | - | 729 |
| *Pyrus pyrifolia* | AP012207.1 | 55 | 159922 | 36.57 | 31.3 | 32.12 | 17.92 | 18.64 | 134 | 26392 | 19237 | 87901 | 70 | 691 | 36 | 458 | 23 | 1208 |
| *Pyrus spinosa* | HG737342.1 | 56 | 159161 | 36.61 | 31.3 | 32.07 | 17.94 | 18.66 | 135 | 24563 | 21416 | 88619 | 64 | 676 | 14 | 166 | 27 | 883 |
| Malus prunifolia | KU851961.1 | 57 | 160041 | 36.56 | 31.32 | 32.11 | 17.91 | 18.64 | 134 | 26359 | 19204 | 88119 | 72 | 648 | 36 | 539 | - | 1223 |

**
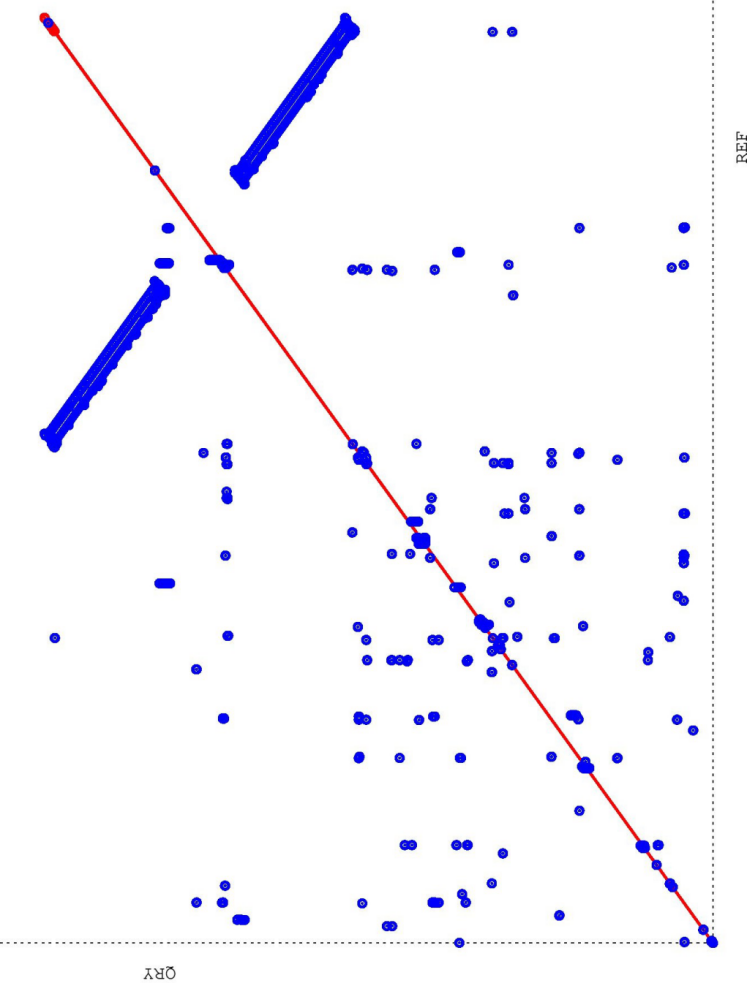
**

**Fig.S1.** Dot-plot comparison showing conserved and inverted regions found in both *Prunus persica* (x axis) and remaining accessions (y axis) cp genomes. Note that the plastome of *Prunus persica* has an inversion region.

**
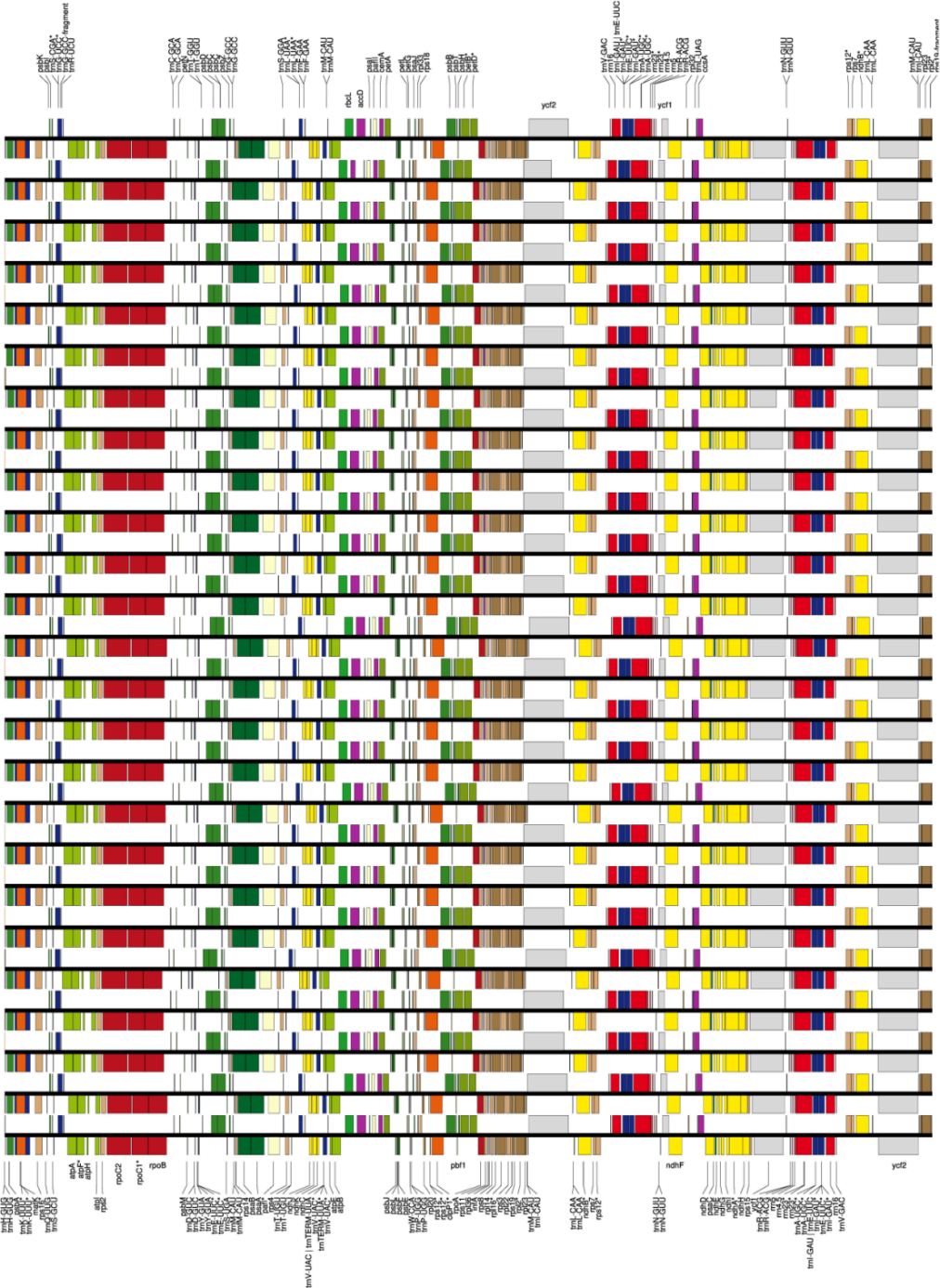
**

**Fig.S2.** Gene maps of the plastomes of the *Prunus* species assembled in this study. Gray shading highlights IR regions with IR boundary shifts. Genes drawn below the line are transcribed, and those drawn above the line are transcribed counter. Genes belonging to different functional groups are colored according to the legend. Asterisks (*) represent intron-containing genes. (F) Representation of the smallest and largest prunus species plastomes studied. Gray regions correspond to the IRs.

**
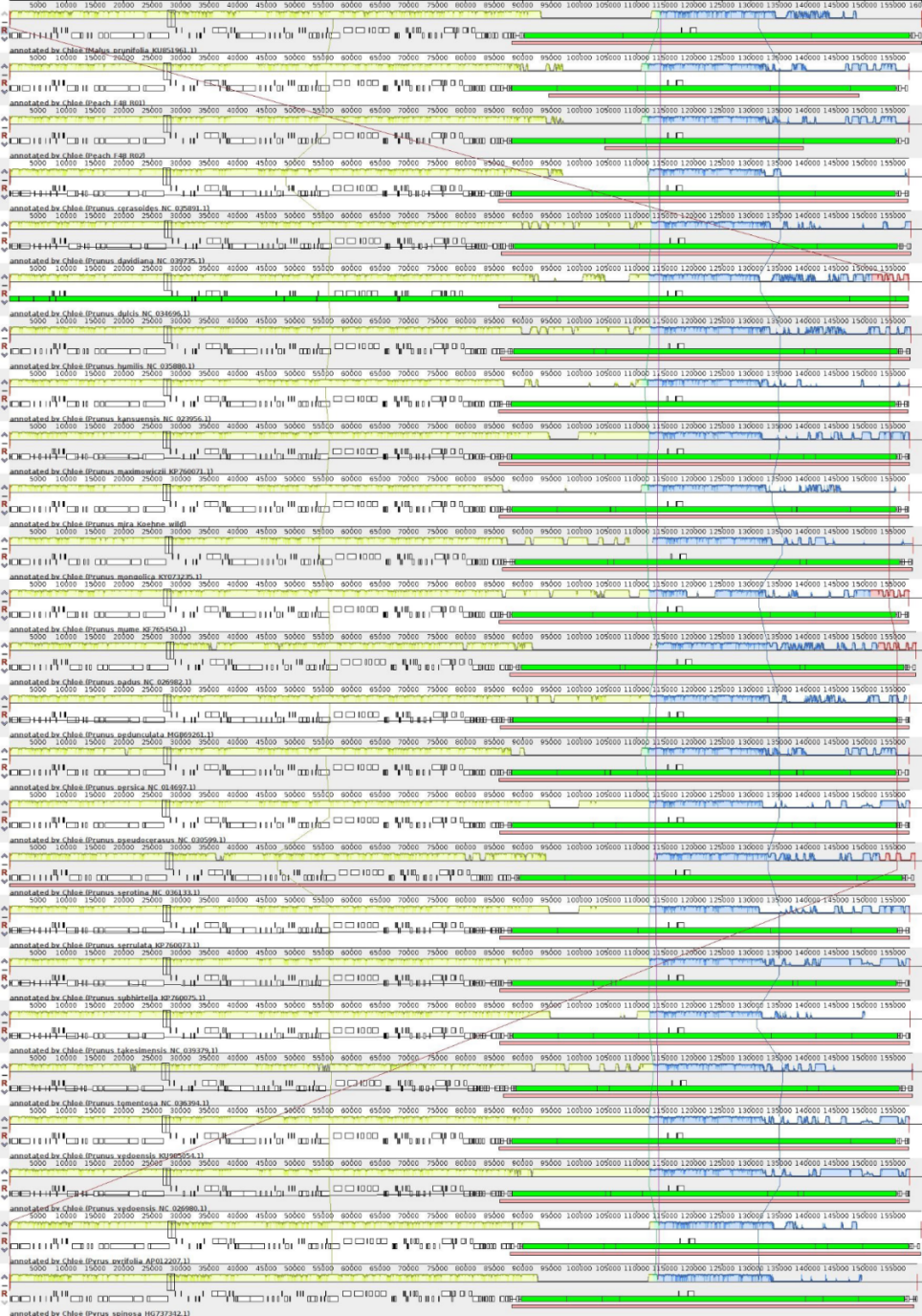
**

**Fig.S3.** Gene rearrangement analyses among *Prunus persica* species by Mauve alignment. The tRNA genes without introns were coded black whereas the tRNA genes containing introns were coded green. The rRNA was coded red, and the protein coding genes were coded white. This figure provides a graphical view of the plastome genome organization of 25 species of *P. persica* and the variation showed by *P. dulcis* and *P. serotina*.

**
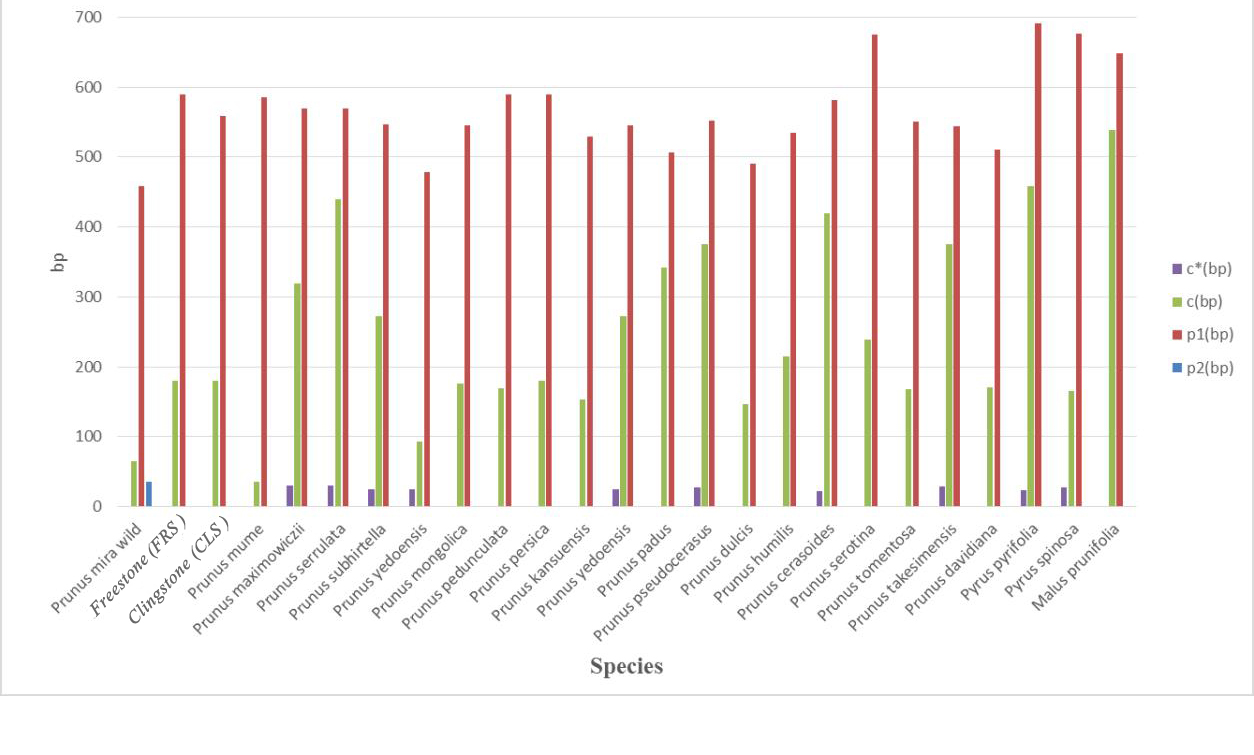
**

**Fig.S4.** Simple Sequences Repeats (SSRs) analysis in *P. persica*. The Distributions of Type P1, P2, C, and C* nucleotide repeats represent mono-, di-, penta- and hexa- were shown.

| 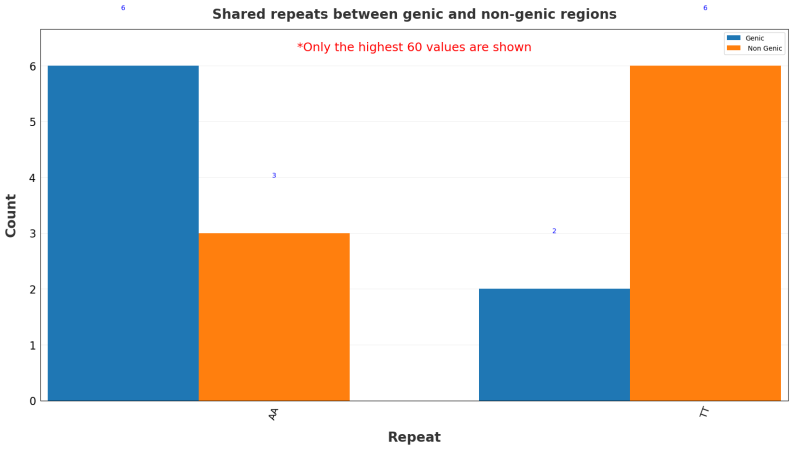  **(A) Freestone** | 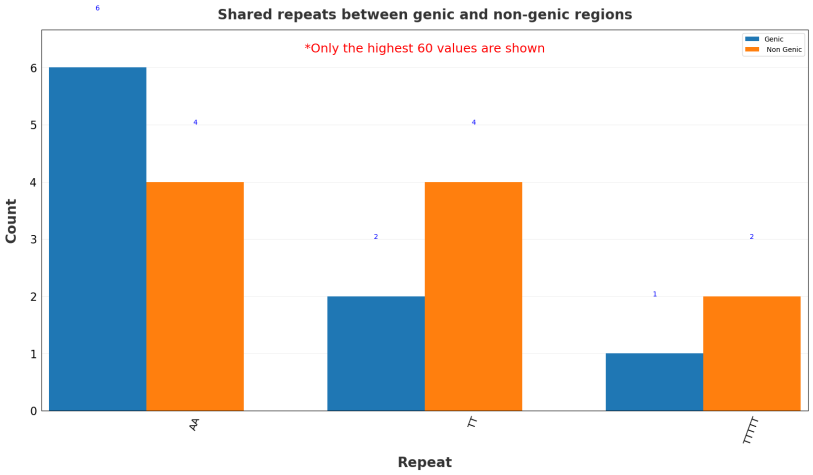  **(B) Clingstone** |
| --- | --- |

# Fig. S5. Common SSR repeats shared between genic and non-genic regions in Freestone (A) and Clingstone (B) cultivars.

| 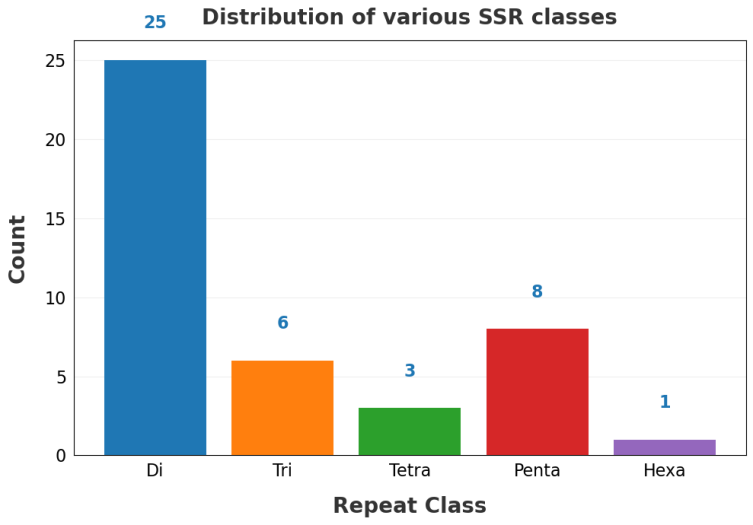  **(A) Freestone** | 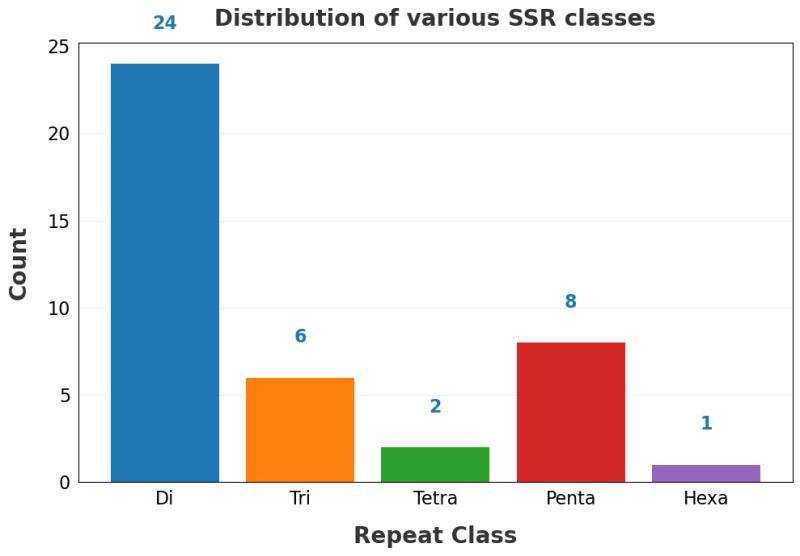 **(B) Clingstone** |
| --- | --- |

**Fig. S6.** Distribution of different SSR classes across the genome in Freestone (A) and Clingstone (B) cultivars

| 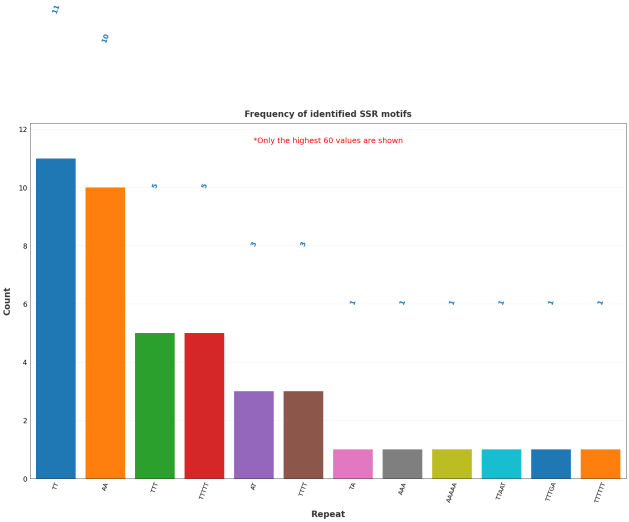  **(A) Freestone** | 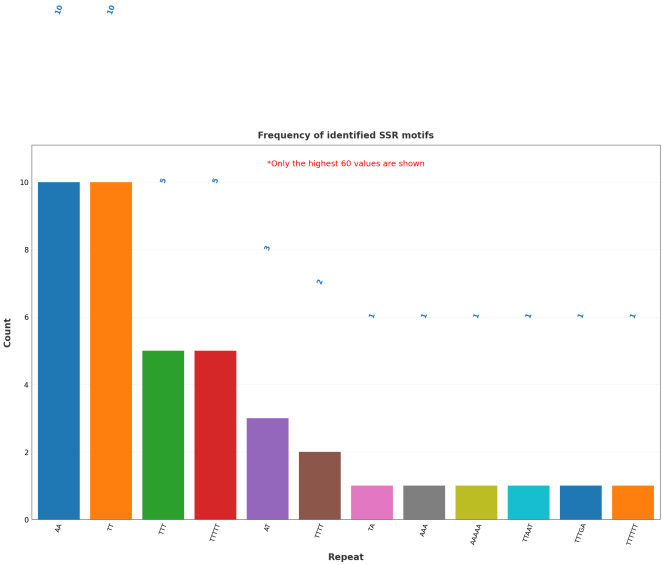  **(B) Clingstone** |
| --- | --- |

**Fig. S7.** Frequency of identified SSR motifs in Freestone (A) and Clingstone (B) cultivars

**Fig. S8.** SSR distribution considering sequence complementarity and strand orientation in Freestone (A) and Clingstone (B) cultivars.

| 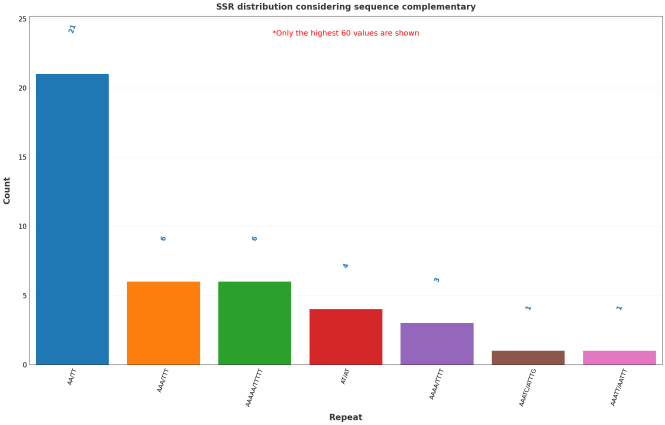  **(A) Freestone** | 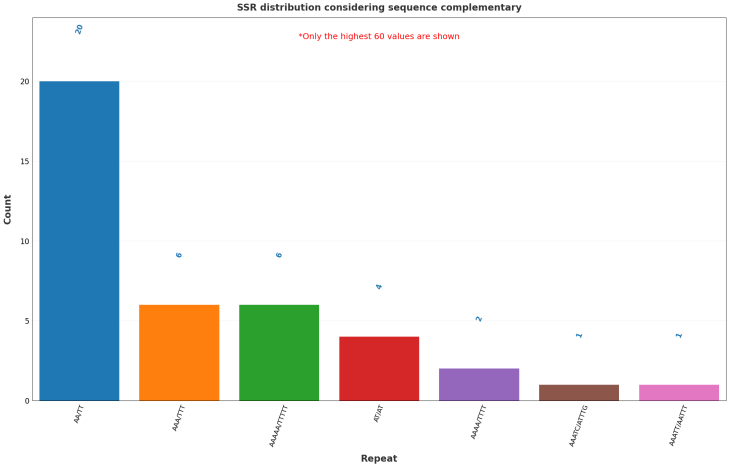  **(B) Clingstone** |
| --- | --- |

**Fig. S9.** Unique SSR repeats identified within genic regions of Freestone (A) and Clingstone (B) cultivars.

| 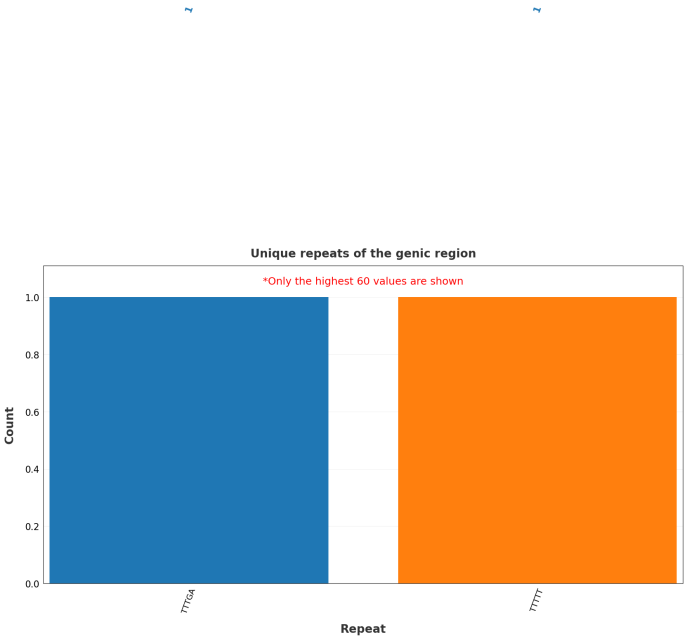  **(A) Freestone** | 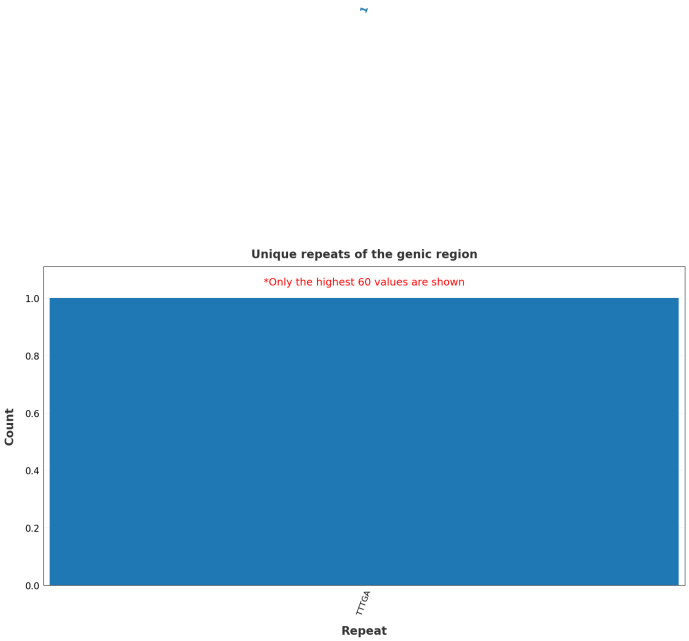  **(B) Clingstone** |
| --- | --- |

| 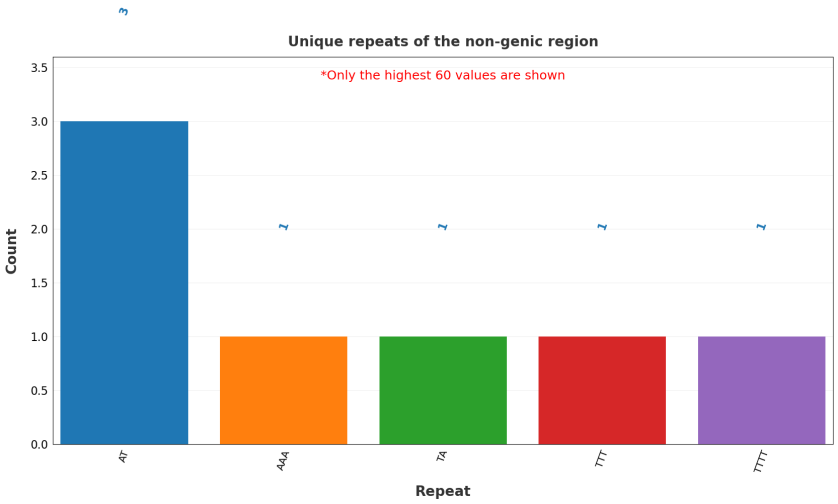  **(A) Freestone** | 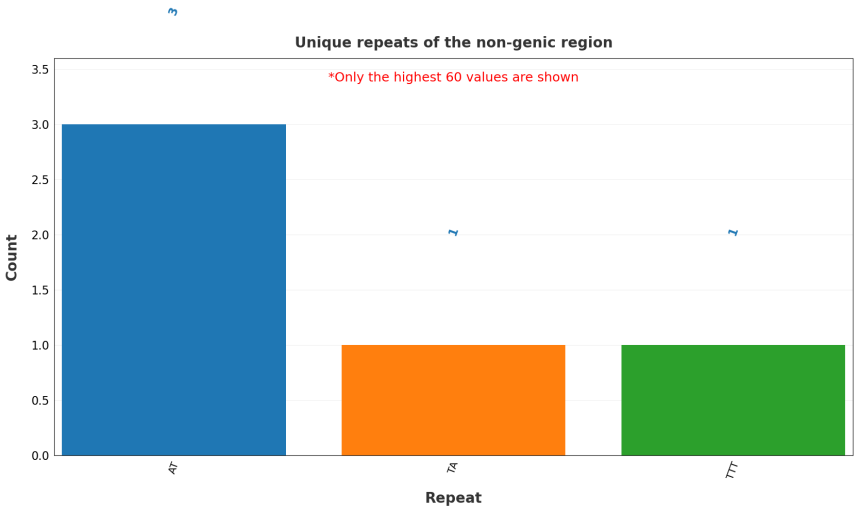  **(B) Clingstone** |
| --- | --- |

**Fig. S10.** Unique SSR repeats identified within non-genic regions of Freestone (A) and Clingstone (B) cultivars.

| 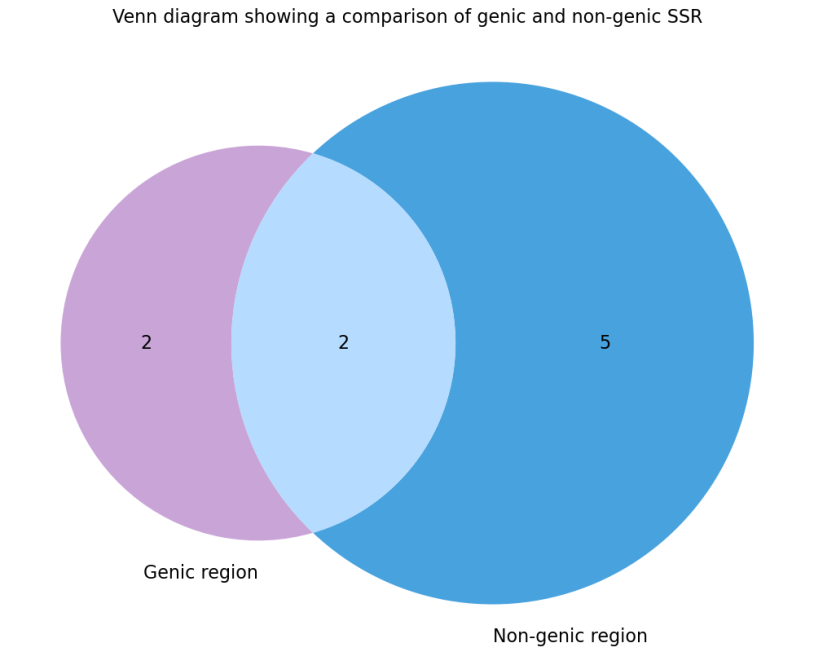  **(A) Freestone** | 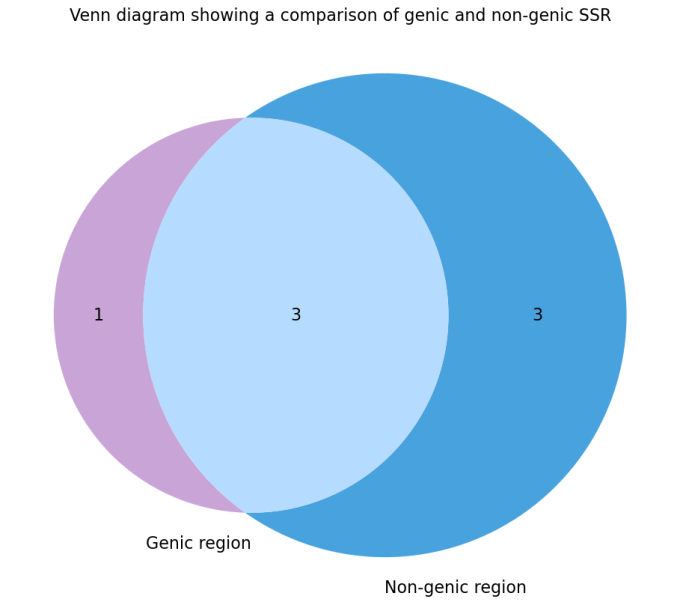  **(B) Clingstone** |
| --- | --- |

**Fig. S11.** Venn diagram illustrating the overlap between genic and non-genic SSRs in Freestone (A) and Clingstone (B) cultivars
